# Combined Orthodontic and Surgical Management for Treatment of Severe Class III Malocclusion with Anterior and Posterior Crossbites

**DOI:** 10.1155/2021/5579077

**Published:** 2021-06-25

**Authors:** Yahya A. Alogaibi, Fahad F. Alsulaimani, Basem Jamal, Rania Mitwally

**Affiliations:** ^1^Orthodontist, Bisha Dental Center, Ministry of Health, P.O. Box 418, Bisha 61922, Saudi Arabia; ^2^Orthodontics Resident, Department of Orthodontic, King Fahad Hospital, Specialized Dental Center, Madina, Saudi Arabia; ^3^Consultant and Professor, Department of Orthodontics, Faculty of Dentistry, King Abdulaziz University, P.O. Box 80209, Jeddah 21589, Saudi Arabia; ^4^Consultant and Associate Professor, Oral & Maxillofacial Surgery Department, King Abdulaziz University, Jeddah, Saudi Arabia; ^5^North Dental Specialty Center, Orthodontic Department, Orthodontist, Ministry of Health, Jeddah, Saudi Arabia

## Abstract

Severe class III malocclusion can be a great challenge, especially in adult patients. This case report describes an adult patient with severe skeletal class III malocclusion and with an obvious maxillary deficiency and mandibular excess causing both anterior and posterior crossbites in addition to a shift in the upper and lower midlines to the left concerning the facial midline. This was complicated by compensatory mechanisms such as the proclination of upper incisors and retroclination of lower incisors. Decompensation of the upper and lower arches was performed combined with upper arch expansion to relieve crowding in the upper arch and correct the posterior crossbite. This was followed by double jaw surgeries, including Le Fort I osteotomy in the maxilla and bilateral sagittal split osteotomy (BSSO) in the mandible. Orthodontic finishing procedures were then used to correct any other dental discrepancies. Remarkable esthetic and functional results were achieved with high patient satisfaction.

## 1. Introduction

Skeletal class III malocclusion is considered to be one of the most difficult cases to treat especially if a severe form is found in adult patients. The deformity was found to be caused by class III malocclusion, which is not always restricted to the dental arches and may involve the total craniofacial complex, and the etiology of this malocclusion is mainly influenced by genetic factors [1]. Most of the patients with class III malocclusions show a combination of both skeletal and dento-alveolar components. These components may act synergistically or in isolation to increase or decrease the severity of the condition [2].

There are three available treatment options for management of skeletal class III malocclusions. These are modification of growth, orthodontic camouflage treatment, or combination of orthodontic treatment and orthognathic surgery. Modification of growth by utilizing orthopedic appliances is only effective for treatment of skeletal class III discrepancies in growing patients [3]. Camouflage orthodontic therapy can be considered to correct mild skeletal class III patients with acceptable profiles. However, management of this problem in adult patients will usually require considering the option of orthognathic surgery, especially if the problem was severe [4, 5].

In severe adult cases with class III malocclusion, a combination of both orthodontic treatment and orthognathic surgery is usually needed to achieve a proper outcome. However, the skeletal type of class III malocclusion usually affects the type of surgery. It was found that 43% of class III cases had only mandibular prognathism with a normal-sized maxilla, while 19.6% had a normal-sized mandible with deficiency and retrognathism of the maxilla, and the smallest percentage (<5%) had both maxillary deficiency and mandibular excess [6].

Surgical intervention in severe class III cases usually involves either maxillary advancement or mandibular setback. These procedures can be performed alone or in combination according to the case severity. Le Fort I osteotomy and/or bilateral sagittal split osteotomy (BSSO) are the most commonly used surgical procedures to treat severe class III malocclusion. In cases requiring orthognathic surgery, a proper treatment plan is required to achieve a good outcome for the treatment together with accurate identification of the patient's expectations [4, 7].

In this case report, we describe treatment of an adult patient with a severe skeletal class III malocclusion using a combination of orthodontic treatment and orthognathic surgery, and orthodontic expansion of the upper arch was performed followed by Le Fort I osteotomy and BSSO to correct this severe malocclusion.

## 2. Diagnosis

**Figure 1:**
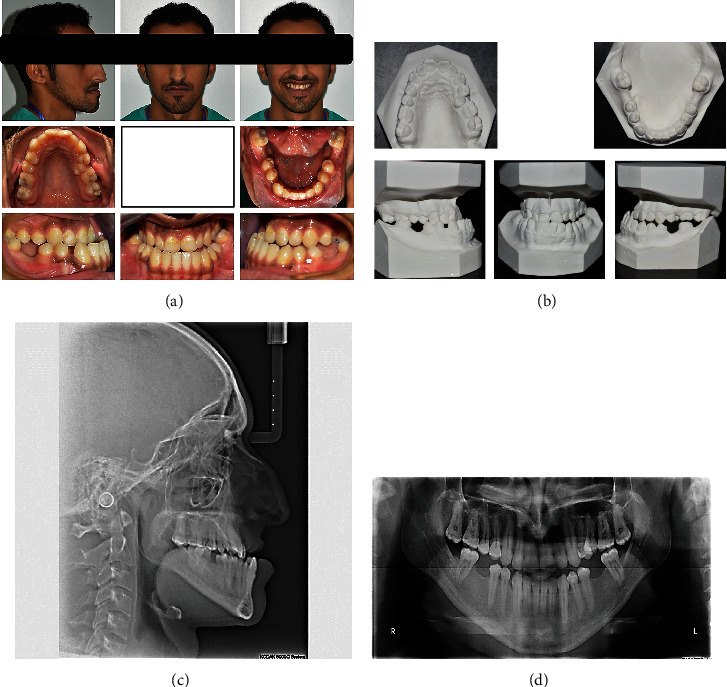
Pretreatment extraoral and intraoral photographs, study models cephalometric radiograph, and panoramic radiographs of the patient: (a) extraoral and intraoral photographs; (b) study models; (c) cephalometric radiograph; (d) panoramic radiograph.

A 31-year-old male patient presented to the orthodontic clinic with a prominent lower jaw. Intraoral examination showed that his oral hygiene was fair, and his teeth showed areas of plaque accumulation and staining. The patient had a history of lower first molar extraction. Facial examination showed that the patient had the typical concave profile of skeletal class III with an increased mandibular plane angle and normal TMJ function. Additionally, intraoral examination revealed a shift in the upper midline to the left by 1.5 mm and a shift to the left of 3 mm in the lower midline in relation to the facial midline. The upper and lower arch relationship showed class III canines on both sides, anterior crossbite with a reverse overjet of −6 mm, and a bilateral posterior cross bite (Figures 1(a) and 1(b)).

Cephalometric analysis for this patient, as shown in Table 1, revealed some anteroposterior skeletal abnormalities including a skeletal class III jaw relationship that was caused by both maxillary deficiency and mandibular excess. Additionally, a normal anterior vertical relationship was found with decreased posterior facial height causing some degree of steepness in the mandibular plane angle. The upper dental arch showed crowding with a palatally erupted upper left second premolar. Upper and lower incisors showed compensatory mechanisms with mild proclination of the upper incisors and moderate retroclination of the lower incisors. Soft tissue analysis revealed the classical appearance of severe class III malocclusion with a retruded upper lip and protruded lower lip (Figure 1(c)). A panoramic radiograph showed condyles that were symmetrical in size and shape without any obvious pathology and missing lower first and third molars (Figure 1(d)).

## 3. Treatment

### 3.1. Treatment Objectives

The proposed treatment objectives were to reinforce and improve oral hygiene, improve the lip position, surgically advance the maxillary basal bone, surgically set back the mandibular basal bone, expand the maxillary arch, correct the anterior and posterior crossbites, correct the midline shifts in the upper and lower jaw, and achieve class I molar and canine relationships and suitable prosthesis for the missing teeth.

### 3.2. Treatment Plan

Surgical and nonsurgical treatment options were discussed with the patient. The nonsurgical approach was based on orthodontic camouflage by extraction of lower premolars followed by retraction with miniscrews combined with upper arch expansion.

Another treatment option was orthodontic decompensation with upper arch expansion followed by double jaw surgery including Le Fort I osteotomy in the maxilla and BSSO in the mandible.

After discussing these treatment alternatives with the patient and explaining the advantages and disadvantages of each option, taking into account both esthetic and functional demands, the second option for treatment was approved.

### 3.3. Treatment Progress

**Figure 2:**
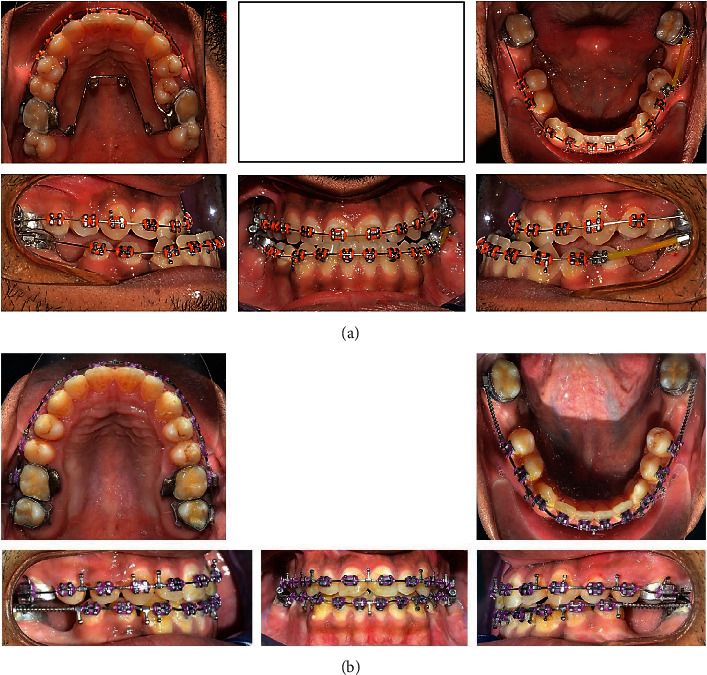
(a, b) Multiple progress photographs.

The treatment was initiated by banding the upper and lower molars together by bonding the other teeth using preadjusted 0.022 × 0.030 inch edgewise Roth prescription brackets. A quad helix was inserted to expand the upper arch. Leveling and alignment to decompensate the upper and lower arches was performed using a progressive increase in wire thickness in the upper and lower arches with Ni-Ti arch wires with bend-back in the lower arch, starting with 0.014″ and reaching 0.019 × 0.025^″^ (Figures 2(a) and 2(b)).

Open coil springs were inserted into the space from the extracted lower first molars to close the spacing anteriorly and to orient the lower second molars in an upright manner. Rigid stainless steel wires (0.019 × 0.025^″^) were used to prepare the patient for surgery. BSSO was performed in the mandible with a setback of 6.9 mm. This was combined with Le Fort I osteotomy in the maxilla with an advancement of 8.5 mm. The patient had also undergone a rhinoplasty procedure 2 months before the orthognathic surgery.

After surgery, finishing procedures were performed to obtain better interdigitation between the teeth. Vertical elastics were applied for 20 hours/day, and they were then reduced gradually until stable occlusal contacts were achieved. A maxillary wrap-around retainer and a fixed canine-to-canine with a wrap-around mandibular retainer were placed. Total treatment time was 2 years and 7 months.

### 3.4. Treatment Results

**Figure 3:**
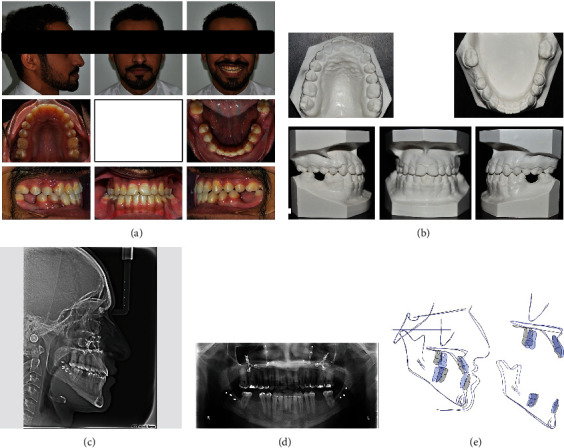
Posttreatment extraoral and intraoral photographs, study models cephalometric radiograph, and panoramic radiographs of the patient: (a) extraoral and intraoral photographs; (b) study models; (c) cephalometric radiograph; (d) panoramic radiograph; (e) cephalometric superimposition.

The extraoral posttreatment photographs showed marked improvement in the facial profile. Chin position was set backward, and the naso-labial angle was decreased to the normal values. The buccal corridors during smiling were reduced with pleasing smile characteristics. The midline shifts in both the upper and lower jaws were corrected together with class I skeletal and dental relationships. Both the posterior and anterior crossbites were eliminated, and normal overjet and overbite were reached (Figures 3(a) and 3(b)).

The cephalometric analysis showed favorable skeletal and dental changes in anteroposterior measurements with minimal changes in vertical proportions. All the teeth showed a normal level of bone with no evidence of root resorption when examined in the panoramic radiograph (Table 1; Figures 3(c)–3(e)).

## 4. Discussion

Combined orthodontic and orthognathic surgical treatment may be required in some cases to achieve the required esthetic and functional results [8]. If there is any skeletal problem that is beyond the limits of camouflage treatment according to the envelope of discrepancy, any trial on dental compensation may reach a reasonably acceptable occlusion using miniscrews as methods of absolute anchorage. However, this requires compromising facial aesthetics because it is not possible to achieve satisfactory facial esthetics using this method. This approach is no longer accepted in modern orthodontics because facial esthetics should never be compromised to reach ideal occlusion [9, 10].

This was why the orthognathic surgical treatment approach was chosen over the camouflage method. Additionally, it is well known that when starting a camouflage treatment approach, it is nearly impossible to go back to a surgical option of treatment using orthognathic surgery because both options are opposite to each other regarding teeth movements and extraction choices. Moreover, surgical correction of skeletal class III malocclusion after combined maxillary and mandibular strategies shows up to be stable for maxillary advancements up to 5 mm and for the correction of presurgical sagittal intermaxillary discrepancies smaller than 7 mm [2, 5, 11, 12].

Orthodontic expansion of the upper arch was performed because the amount of crowding and maxillary constriction was not severe. Although the bilateral posterior crossbite was obvious, this crossbite was partially caused by the backward position of the maxilla and the forward position of the mandible, which caused a wider molar area of the mandible to be occluded with the narrower premolar area of the maxilla, which suggests a severe maxillary deficiency. Moreover, by calculation of the needed space to relieve the crowding in the upper arch [13, 14], we found that expanding the upper arch from both the intercanine and intermolar area was sufficient to relieve the crowding without requiring extraction.

The most suitable appliance that was found to achieve a differential expansion at the intercanine and intermolar area was the quad helix. Quad helix was selected as an effective expansion method for this patient because of its ability to deliver a constant physiologic force to achieve the required expansion and prevent buildup of excessive forces on the maxillary complex [15, 16].

Surgically assisted rapid maxillary expansion was another option for expanding the upper arch, which has the ability to produce more skeletal expansion in adult patients with a completely fused midpalatal suture. However, root resorption and possible trauma to the condyles was reported in some cases that used this expansion technique. A significant amount of relapse was also reported [17, 18].

Finally, the rationale for selecting an upper wraparound retainer was based on its ability to maintain the achieved transverse correction by expansion. However, fixed canine-to-canine with a wraparound mandibular retainer was used to retain the incisor position and to prevent loss of the space from the extracted first molars [19, 20].

## 5. Conclusion

Severe skeletal class III malocclusion cases can sometimes be impossible to handle with orthodontic treatment alone, especially in adult patients, because orthodontic camouflage can produce less than optimal results with inadequate patient satisfaction. Orthognathic surgery may be the only reliable and evidence-based approach to achieve stable esthetic results. However, applying conservative orthodontic solutions to relieve crowding with an effective decompensation of the arches should be considered as a first choice during treatment planning. This conservative approach can reduce the treatment time and minimize the possibility of patient burnout during the course of treatment.

## Figures and Tables

**Table 1:** Cephalometric analysis.

		Measurement	Mean (±Sd)	Patient	
				Initial	Final
	Ant-posterior	SNA (°)	82° (±3.3)	76.2	80
		SNB (°)	80° (±3.1)	82.1	79
		ANB (°)	2° (±1.7)	-5.9	1
		Wits (mm)	*M* = −1.17 (±1.9) *F* = −0.10 (±1.77)	-16.1 mm	-2
		Angle of convexity NA-APg (°)	0° (±5.1)	-11	-1
		A-B plane AB : NPg (°)	-4.6° (±3.7)	10	-2

	Vertical	MP (Go-Gn) : SN (°)	32° (±3.5)	44	43
		MP (tangent lower border) : FH (°)	21.9° (±3.2)	31	31
		Pg : NB (mm)	4 (±2)	-1.5 mm	3.6 mm
		*Y*-axis (SGn : FH)	59.4° (±3.8)	69	70
		LAFH (ANS to Gn ÷ N to Gn)	.57 (±0.02)	56%	56
		OP : SN (°)	14° (±4.1)	21.8	21.1

Dental		U1 to palatal plane (°)	109° (±6)	102.6	105.9
		U1 to NA (°)	22° (±6.1)	26.4	27.2
		U1 to NA (mm)	4 (±1.2)	5 mm	6 mm
		L1 to NB (°)	25° (±4.5)	15.1	15.1
		L1 to NB (mm)	4 (±1.5)	2 mm	3 mm
		U1 to L1 (°) (Avg. Downs and Steiner)	131.7° (±6.5)	144	137
		L1 : APg (mm)	1 (±2)	7 mm	1 mm
		FMA (°)	25° (16-35)	31	31
		FMIA (°)	65° (60-75)	80	75
		IMPA (°)	90° (85-95)	69	74

Soft tissue		Facial angle (FH : N'Pg') (°)	90-92°	94	92
		Nasolabial angle (°)	90-110°	113	88
		Esthetic plane (E-line)–upper lip	-4 mm	-13 mm	-6 mm
		Esthetic plane (E-line)–lower lip	-2 mm	0 mm	-3 mm

## Data Availability

No data were used to support this study, only the case which has been done in the Orthodontic Department, Dentistry College, King Abdulaziz University.
